# Global repeat discovery and estimation of genomic copy number in a large, complex genome using a high-throughput 454 sequence survey

**DOI:** 10.1186/1471-2164-8-132

**Published:** 2007-05-24

**Authors:** Kankshita Swaminathan, Kranthi Varala, Matthew E Hudson

**Affiliations:** 1 Department of Crop Sciences, University Of Illinois, Urbana, IL 61801, USA

## Abstract

**Background:**

Extensive computational and database tools are available to mine genomic and genetic databases for model organisms, but little genomic data is available for many species of ecological or agricultural significance, especially those with large genomes. Genome surveys using conventional sequencing techniques are powerful, particularly for detecting sequences present in many copies per genome. However these methods are time-consuming and have potential drawbacks. High throughput 454 sequencing provides an alternative method by which much information can be gained quickly and cheaply from high-coverage surveys of genomic DNA.

**Results:**

We sequenced 78 million base-pairs of randomly sheared soybean DNA which passed our quality criteria. Computational analysis of the survey sequences provided global information on the abundant repetitive sequences in soybean. The sequence was used to determine the copy number across regions of large genomic clones or contigs and discover higher-order structures within satellite repeats. We have created an annotated, online database of sequences present in multiple copies in the soybean genome. The low bias of pyrosequencing against repeat sequences is demonstrated by the overall composition of the survey data, which matches well with past estimates of repetitive DNA content obtained by DNA re-association kinetics (Cot analysis).

**Conclusion:**

This approach provides a potential aid to conventional or shotgun genome assembly, by allowing rapid assessment of copy number in any clone or clone-end sequence. In addition, we show that partial sequencing can provide access to partial protein-coding sequences.

## Background

Genome sequencing has historically been accomplished by fragmenting genomic DNA, amplifying the fragments clonally using bacteria, and sequencing the amplified clones [1]. Although this method has improved to the extent that much larger genomes can be sequenced, and some of the intermediate cloning steps can be circumvented [2,3], practically all genome sequence until very recently has been generated by the Sanger method. Given the costs of Sanger-based genome sequencing and surveys, significant amounts of genomic information for most of the 129,293 eukaryotic species listed in the NCBI taxonomy database [4] are unlikely to be available for some time. Soybean [*Glycine max *(L.) Mer], which is the subject of this study, has an existing but incomplete genome project. However, many crop plants, plant pathogens, endangered species and species of evolutionary interest have little or no available genome data. Recently developed microbead technologies capable of sequencing hundreds of thousands of DNA molecules in parallel provide a way to obtain genomic information from these species for reasonable cost, and without any bacterial cloning step. The method used here, 454 pyrosequencing, uses pyrophosphate release as a method for detection of base incorporation [5-7]. Pyrosequencing has been used before to genotype SNPs in a polyploid plant, potato. However, the technology used [8] relied on known primer sequences, greatly limiting the utility of the method for *de novo *sequencing. The 454 pyrosequencing method uses randomly sheared DNA, has no requirement for known primer sequences (making it suitable for *de novo *sequence surveys), and makes sequence data faster and cheaper to obtain than Sanger-based methods. However, the accuracy and read length of the method as used here is generally inferior to Sanger-based sequencing of small clones [9].

The first step in characterizing large genomes has frequently been a genome survey, often using end sequences of Bacterial Artificial Chromosome (BAC) vectors [10,11]. Such a survey gives important information about common repeat sequences, allows the generation of some genetic markers and helps determine the feasibility of building a BAC tiling path. Such surveys are limited, however, as representation of cloned sequences is likely to be somewhat skewed towards those that can be successfully propagated in bacterial vectors [12]. Here we describe a method for performing high coverage, inexpensive and detailed genome surveys without the necessity of cloning, bacteria or vector libraries. The 454 pyrosequencing method described by Margulies et al. [9] allows access to randomly placed, short sequences in large numbers, without the generation of bacterial vectors or a cloning step. Since 454 pyrosequencing produces relatively short reads, without paired end information, it is currently unsuitable for *de novo *sequencing of eukaryotic whole genomes. However, a high-coverage genome survey using this method can potentially deliver invaluable data about the makeup of a genome, quickly and at relatively low cost. In particular, the identification of sequences present in many copies per genome (essential in order to generate a unique tiling path for a structured sequencing approach) is straightforward.

The soybean genome is relatively well-characterized, and significant progress has been made towards its completion. A survey of BAC clone ends has been performed at relatively low coverage on the soybean genome [10], and extensive sequencing of soybean ESTs has been performed [13]. However, a complete physical map is not yet available, and the amount of soybean genomic sequence in the public domain is still somewhat limited, although now growing rapidly. The survey described here provides further information about the makeup of the genome of this crop of great commercial importance.

## Results

### Sequencing of 78 million bases of random short reads from the soybean genome

Genomic DNA was extracted from purified nuclei isolated from leaves of soybean cv. Williams. The DNA was randomly sheared, and sequenced using the 454 pyrosequencing method [9]. This resulted in 717,383 successful sequence reads, together with phred-equivalent quality (Q) values [14]. Mean read length of these filtered, trimmed reads was 109.5 base pairs (bp), with a total of 78,535,105 bp of sequence generated. The soybean haploid genome size has been estimated at 1,115 million base pairs (Mb) [15], therefore the filtered, trimmed reads used in this sequence survey represent an estimated 7% coverage of the soybean genome.

The 103-Kb region surrounding the *CHS *locus of soybean has been extensively characterized [16]. We utilized the sequence of this region to probe the genomic distribution and accuracy of the genomic survey sequences. Using BLAT [17], 102 reads with 95% or higher identity across 98% or more of the read to this validated sequence were identified. These reads represent 10,542 base pairs of sequence with an overall 97.7% match to the validated sequence, hence there is a minimum estimated error rate of 2.3%. The presence of slightly more than the expected number of matching reads within the pyrosequencing dataset provides evidence that the estimated genome size of soybean [15] is approximately correct.

### Analysis of high-abundance sequences in soybean

Repetitive sequences can confound both common methods for *de novo *genome sequencing: conventional, tiling-path based assembly strategies and shotgun genome sequencing approaches. Consequently, we aimed to develop accurate repeat detection methods and comprehensive cataloging of repetitive sequences.

Using the annotated TIGR databases [18] from multiple species, we are able to estimate the genomic copy number of all of the repeat classes in the TIGR collection. These repeats may be detected either through similarity to *Glycine *or to repeats known from other plant genomes, including the completed genomes of *Arabidopsis *and *Oryza*.

The TIGR plant repeat database is composed largely of transposable element sequences and noncoding RNA genes, and as with any database using incompletely sequenced genome data, it is incomplete. Satellite sequences such as those detected in the assembly of our own soybean repeat database are under-represented in the TIGR repeat database, despite their presence in GenBank, and the types of repeat and organisms of origin of the sequences vary.

**Figure 1:**
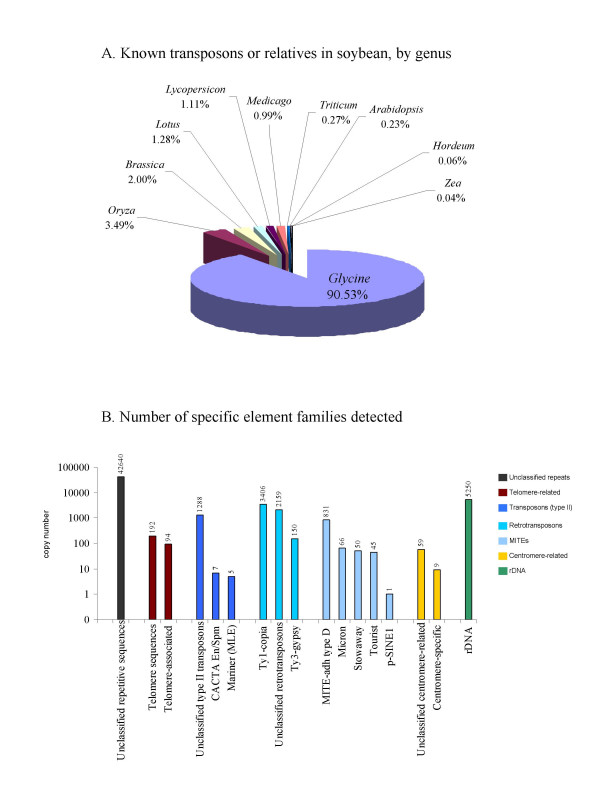
**Comparison of sequence survey data with soybean and other plant repeat databases**. A) Distribution of hits to plant repeat databases, by genus. Raw reads were matched using BLAST (blastn) to the TIGR plant repeat databases and the top significant (1E-6) hit recorded. Percentages represent the percentage of reads with hits to sequences from a particular organism with respect to all reads with hits to the TIGR repeats. B) Distribution of hits to plant repeat databases, by class of repetitive element Raw reads from the genomic sequence survey were matched to the combined plant repeat databases as for (A), and the class of repetitive element for the top hit was used to show the relative abundance of different classes of repetitive elements. This gives an estimate of the relative frequency of these families in the soybean genome. Retrotransposons and rDNA are the most common classes of repeat. See Additional File 1 for common repeat sequences not included in the TIGR database.

For each of the 717,383 reads, we searched for a significant (e < 1E-6) BLAST (blastn) sequence match to the TIGR plant repeat database, which is organized both by species of origin and class of element. Figure 1A shows the percentage of reads with top hits that matched each species represented in the TIGR database. The most abundant matches are those to repetitive elements already known to exist in *Glycine max*. Since the most abundant sequences in soybean are also the most likely to be well-characterized in this organism, this was an expected result. However, the database contains other legume repeat sequences: 64 sequences from *Lotus *species, 128 from *Medicago *species, as well as 130 from *Glycine *species. We were surprised that the *Lotus *and *Medicago *matches were not more abundant. We speculate that this may be because the *Lotus, Medicago *and soybean sequences are mostly related, and hence the reads with a match to legume repeats generally have their best (lowest blastn expect value) match to the *Glycine *sequences. Note that most of the *de novo *detected repeats from our survey, including the SB92 and STR120 satellites (present in the GenBank nucleotide (nt) database) and many retrotransposons described in Additional File 1 (many of which are not present in nt), were not present in the TIGR database.

Relatively few reads matched the repeat database for Arabidopsis. Most of the reads similar to repeat sequences from other plants (i.e. elements that were previously unknown in *Glycine*) had their most significant similarity to sequences from *Oryza *or from *Brassica. Brassica *is more closely related to soybean than *Oryza*, but has been the subject of very limited genomic sequencing, while *Oryza *has been completely sequenced but is much more distantly related to soybean than *Brassica *or *Arabidopsis*. The result that there are more similarities between *Glycine *repeats and *Oryza *repeats, and between *Glycine *and the known repeats from *Brassica*, than to *Arabidopsis *was therefore unexpected.

This analysis also allows description in broad terms of the abundance of transposable element sequence families in *Glycine*, given the presence of related sequences in the database used for comparison. Regardless of species of origin, a family was assigned to each soybean sequence read with a significant (< 1E-6) BLAST (blastn) hit to the TIGR plant repeat database. Figure 1B gives an overview of the repeat composition of the soybean genome and an expected minimum genome copy number for each element type found in the database used. Again, we cannot expect the reference database to be in any way complete, so no conclusions regarding absent sequences can be made. We estimate that the soybean genome contains a minimum of just over 8,000 transposable elements of types named and present in the TIGR repeat database; many more "unclassified repetitive" sequences that have similarity to sequences in this dataset (at least 42,000) are present. One result that arises from this analysis is that while retrotransposons are common in the soybean genome, Type II transposons are likely to be relatively rare (several examples are present in the database, but few match our soybean survey). More noteworthy was that no hits were observed to MULE (MUtator-Like Element) transposons in the TIGR collection. It is likely therefore that soybean MULEs are sufficiently divergent in sequence from any MULEs in the TIGR repeat database that they are not detected by a BLAST (blastn) search. Conversely, while MITEs (Miniature Inverted-repeat Transposable Elements) were not previously present in the TIGR soybean repeat database, sequences with hits to MITE elements from other organisms in the TIGR plant repeats are present in many different classes, indicating the presence of several MITE families in the soybean genome.

### *De novo *detection of abundant sequences in the soybean genome

Identification of repetitive elements using high throughput survey sequencing is not limited to sequence homology searches to known repeats from other genomes. Repeats can also be identified based on their over-representation in the data set. By clustering non-cognate, overlapping DNA sequence fragments using phrap [19] we were able to identify a comprehensive set of sequences present in many copies in the soybean genome. The expected number of cognate contigs obtained by sequencing 7% of a non-repetitive genome was calculated according to Lander and Waterman [20]. Since 7% of the genome was sequenced, assembly into non-cognate contigs allows detection of sequences present in 14 copies or more per genome. The observed excess of overlapping sequences from phrap assembly was used to estimate the relative amount of repetitive DNA present in 14 or more copies in the soybean genome. These calculations are summarized in Table 1. Note that most (81%) of the predicted repetitive sequence is in contigs that contain more than seven reads, all of which are likely non-cognate since none are expected to be generated by chance from non-repetitive DNA. In total, approximately 41% of the total reads in the survey (293,889 out of 717,383) were found to form contigs only expected to assemble if the underlying sequence is present in multiple copies [Table 1]. We thus estimate that 41% of the soybean genome is present in more than 14 copies per haploid set. Most of these repeats (comprising an estimated 33% of the soybean genome) are present in 100 or more copies. This estimate is in strong agreement with past DNA re-association kinetics (Cot measurements), which predict that 30–45% of the soybean genome consists of highly repetitive DNA, with the total repeat content in the range of 40–60% [21,22]. However, unlike Cot measurements, this method gives access to the underlying sequence of the detected repeats.

**Table 1:** Repetitive sequences in the soybean genome quantified using the difference between the contigs produced by an assembly algorithm with conservative parameters, and the predictions of the Lander-Waterman model for sampling a completely non-repetitive genome

Number of reads in contig	Predicted by model	Observed number of contigs	Repetitive reads (Observed-predicted)
2	41,126	42,221	2,189
3	2,511	9,742	21,693
4	153	3,498	13,379
5	9	1,646	8,183
6	1	937	5,619
7	0	634	4,438
> 7	0	4,213	238,389
			
			total 293,890

Our assembly yielded 20,670 predicted repetitive contigs (contigs assembled with three or more reads per contig). The Missouri repeat database [23] contains 348 sequences, the soybase.org collection [24] 5,010 repeats, and the TIGR *Glycine *repeat database [18] 130 sequences. Using BLAST with an e value cutoff of 1E-6, we determined that our repeat database contains 19,274 repeats with no similar sequences in the Missouri collection, 16,261 repeats with no similar sequences in the soymap.org collection, and 20,124 with no similar sequences present in the TIGR *Glycine *repeat database (although more reads from our survey show significant similarity to TIGR repeats from other organisms, as discussed above).

The most abundant repetitive sequences which assembled into higher-order sequence structures were the 92 bp repeat family (GI:402616); these are present in multiple distinct contigs of higher-order repeats [Additional File 1]. In total, 26,714 reads, or 3.7% of the soybean genome sequence, are contained in SB92-like higher-order repeats. However, the published SB92 repeat sequence, which is found in centromeres as well as other genomic locations in the annual soybeans [25] matches only 4,567 reads by BLAST (blastn with e < 1E-6). This indicates the variability of the repeat units within the higher-order contigs, many of which are not close enough to the published, canonical SB92 sequence to match it in our BLAST search. This is consistent with observations [25] that the SB92 repeat has a high level of sequence diversity. A total of 51 contigs contain SB92-like sequences (the most abundant are shown in Additional File 1), but these sequences do not assemble into a single unit. This indicates that distinct subtypes and higher-order structures of this satellite sequence are present in the soybean genome.

In addition to a large number of satellite repeats, we detected novel transposable elements (not detected by BLAST (blastn) comparison to the TIGR repeat database above, presumably because no similar elements are present in that collection). These elements correspond to 25 different classes, including both Type I and Type II transposons. In support of the hypothesis that MULEs do in fact exist in the soybean genome, we detected two MULEs in our *de novo *soybean repeat assembly. These MULE elements have contig IDs 39304 (estimated approx. 25 copies/genome) and 66822 (estimated approx. 40 copies/genome).

The 40 most abundant sequences detected by assembly of the survey data, the number of reads encoding each, and the percentage of the genome that each is predicted to represent, are summarized in Additional File 1. Note that the list is dominated by SB92 repeats, STR120 satellites and calypso/diaspora type retrotransposons. The full list of assembled repeats is available online [26]. An estimated genomic copy number is given, based on the size of the contig and the number of reads it contains (see Methods section). However, we are unable to determine from our survey whether these sequences actually occur in the stated copy number as contiguous units, or whether fragments of these sequences may occur in separate locations. The copy number is our best estimate of the relative abundance of these high-copy-number sequences.

We have compiled and curated the multiple copy sequences discovered using the above sequencing approach and phrap assembly into a soybean repeat database, available from the authors' web site [26].

### Using survey data for genomic copy number analysis

**Figure 2:**
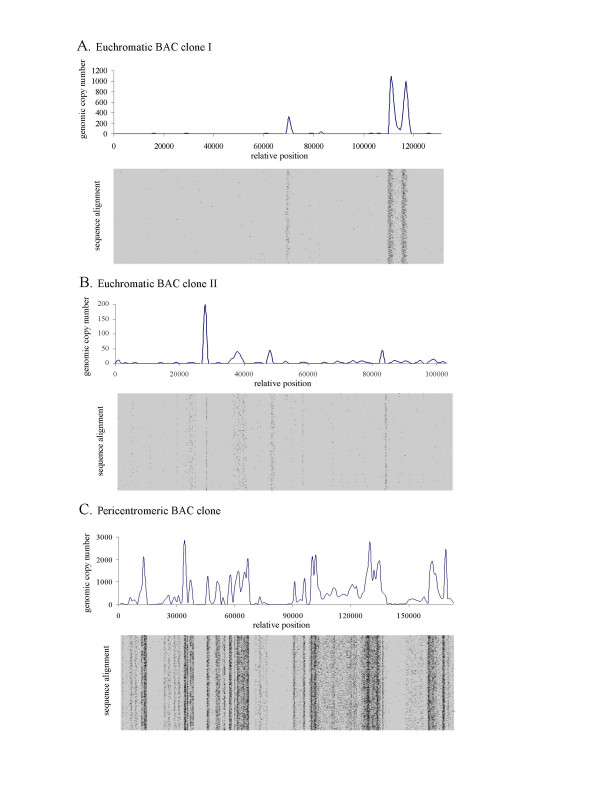
**Alignment of sequence survey reads to BAC clones**. The figure shows a graphic of the alignment of survey reads using BLASTZ to three genomic Bacterial Artificial Chromosome (BAC) sequences of soybean DNA, and estimation of copy number. Copy number was estimated according to the number of sequence survey reads aligning to each 1 kb window of the BACs. The alignment represents the superposition of identical or closely related sequences on the BAC sequence, in order to visualize the individual reads showing regions present in many copies per genome. The BAC sequences were: A) The euchromatic BAC described by Clough *et al*.(20); B) the euchromatic BAC GM_WBb0098N11; C) the BAC GM_WBb0078A23 from a heterochromatic region

Assuming that sequences in our genomic DNA survey are sampled without bias for particular sequence types, the genomic dataset provides a method of estimating the copy number of any genomic sequence. Since the reads are shorter than Sanger sequencing reads, the same amount of sequence provides a higher sampling rate throughout the genome. A 7% coverage survey with 109.5 bp reads provides 6.25 reads per 10 kb of single copy sequence. By comparison, a Sanger-based survey with 700 bp reads, and with no read pairing, would have a sampling rate of 1 fragment/10 kb at 7% coverage. Since most Sanger sequencing is done using read pairs, this would further reduce the effective sampling rate to one read pair (~1,400 bp) per 20 kb of genomic sequence. Hence, the 454 pyrosequencing survey data can be used to estimate the copy number of any 10 kb window of genomic sequence with relative accuracy, as well as detect high-copy-number sequences accurately across much shorter windows. We utilized the sequence of the CHS region, used earlier to probe the accuracy of the genomic survey sequences, to demonstrate the utility of this approach to detect repeats. The CHS sequence is extensively annotated at the gene level but not previously annotated for noncoding repetitive regions, since no databases of repeats were available to the authors of that study [16]. The survey reads with substantial identity to this region were identified with BLAT, then assembled to the genomic sequence backbone, and further inconsistent matches were excluded using a blastz [27] alignment (using default options for gap penalties, MSP and gap thresholds, chaining and word size). The resulting alignment consists of closely related, but not necessarily directly cognate sequences, since repetitive sequences from other genomic regions are intended to assemble with the repetitive regions in the query sequence, allowing them to be visualized. Since approximately 7% of the genome was sequenced, approximately 7% coverage is expected for single-copy sequences, and higher coverage indicates repeated regions. Expected copy number can thus be calculated from the coverage of each sequence window across the alignment. Many regions would be expected to be present in two or more copies as a result of the history of the soybean genome, which involves relatively recent duplication [28]. Using the laj viewer [29] and scripts written in-house [26] (source code available on request from the authors), we created graphical views of the alignment. The resulting graphic [Figure 2A] shows the superimposition of the microbead reads matching the BAC sequence containing the 103-Kb region surrounding the *CHS *locus. This clearly defines regions of the BAC that are present in multiple copies per genome, and shows estimated copy number of these regions. We repeated this analysis with two more BACs, GM_WBb0078A23 and GM_WBb0098N11, available from the soymap.org site [24]. Neither BAC had any associated annotation at the time of writing. The BAC clone GM_WBb0078A23 is derived from a pericentromeric region, whereas GM_WBb0098N11 is from a euchromatic region of the genome [S. Jackson, Purdue University, personal communication]. The two euchromatic BACs [Figure 1A and 1B] have a similar appearance – low or single copy regions form most of the sequence, and they are interspersed by sequences that are found in tens, hundreds or thousands of copies, such as stretches of satellite repeats or transposable elements. In contrast, the pericentromeric sequence is composed to large extent of sequences that are present in hundreds or thousands of copies [Figure 1C]. Note that some regions of the pericentromeric BAC are estimated to be present in few copies, possibly as few as one copy, per genome. This approach is thus potentially useful for detecting unique, possibly genic regions within sequences that are largely repetitive.

In addition to developing a database of repetitive sequences, we have developed a graphical tool for alignment of any sequence to the raw read data, to allow the detection of repetitive regions. The whole-genome copy number of sequence fragments from BAC or other genomic clones can be assessed using the search and alignment viewer, which is available at the authors' web site [26].

### Higher-order structure of repeats within satellite sequence

We were able to assemble some of the smaller, tandem satellite repeats detected in our survey (for example, the previously known STR120 repeat) into non-cognate but deeply sequenced higher-order units using the data from our high-coverage survey. Other sequences, such as retrotransposons, were assembled into a single unit. In order to validate the assembly of selected assembled abundant sequences, both single unit and higher-order satellite, we used PCR amplification to determine the presence of a block of the predicted size in the genome, and used conventional sequencing to confirm the identity of the fragment. Three such amplicons, two higher-order satellite sequences and one putative retroelement, were amplified from genomic DNA to provide validation of the non-cognate assembly data. The PCR fragments are shown in Additional File 2. The fragments from Contig 80285 (gag-pol) and Contig 80369 (STR120 repeat) were cloned and the fragment ends sequenced from vector primers. The fragment from Contig 80374 (another STR120 higher order repeat) was refractory to cloning, and was sequenced in part directly from the gel-purified PCR product using the amplification primers. All sequences matched the contig assembled from the 454 sequence survey, with some base mismatches. Fragments 1 and 2 matched their predicted contigs with > 95% sequence identity across the sequenced length in a global pairwise alignment. No sequence was 100% identical to the predicted contig, probably due to the degeneracy between similar repeats expected *in vivo*. Fragment 3 was more divergent to our predicted sequence, with a BLAST match at > 95% identity but an overall identity of 87% to the predicted contig in a global pairwise alignment. We attribute this to a higher level of degeneracy within this higher-order repeat family *in vivo*, with the cloned fragment being divergent from the most common sequence predicted by the genome survey.

### Analysis of conceptual translations from randomly generated genomic reads

**Figure 3:**
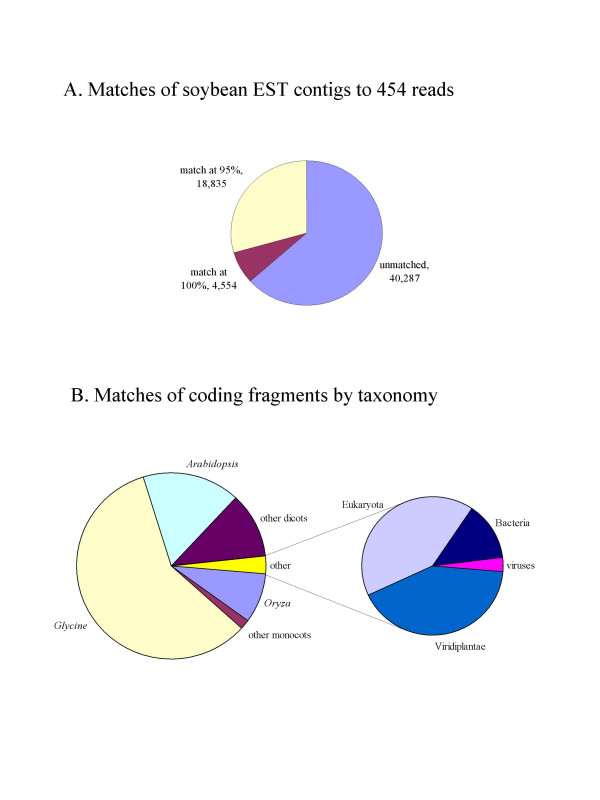
**Annotation of protein ORFs with hits to public database**. A) Proportion of EST clones from the Glycine Max Gene Index (GMGI) matched by 454 reads at 95% and 100% sequence identity (using BLAST with e < 1E-6). The total number of sequences matching at 95% or higher identity is 37% of total EST clones. Note that few sequences match at 100% identity due to the error rate of the 454 pyrosequencing used for this study.B) Coding fragments discovered within the short reads (with e values to the GenBank protein (nr) database < 1E-6), and their closest protein-level sequence hit by taxonomy of the source organism of the database sequence.

The average read size of our survey was 109.5 bp, giving a maximum average open reading frame size of 37 amino acids. Consequently, reads that are derived completely from exonic sequence are a potential source of partial protein sequence. The GMGI database v. 12.0 was used to estimate our survey's coverage of coding regions of the genome [30]. This contains 63,676 sequences with an average length of 594 base pairs. A BLAST (blastn) search was performed with each GMGI sequence as a query and the survey reads as a database, with an expect value cutoff of 1E-6. Figure 3A shows the number of soybean ESTs with 95% or higher nucleotide level sequence matches to the raw reads, 23,389 of 63,676, or 37%. Since seven percent of the genome was covered with average 109.5 base pair reads, we expect approximately 37% of the ESTs known from soybean to have hits to the genomic reads. This concordance provides further evidence of the unbiased random sampling of genomic sequence by our sequencing method, and further evidence that the genome size estimate of 1,115 megabases of Arumaganthan and Earle [15] is approximately correct.

In addition to sequences that have hits to the GMGI EST collection, a number of reads contained open reading frames with BLAST hits to known proteins from other organisms, but no hits to soybean ESTs or other soybean sequences. Figure 3B shows the distribution of coding fragments with an open reading frame giving a 1E-6 or lower BLAST (blastp) e value to the nr database, and the taxonomy of the organism from which the closest sequence in that database was generated. This demonstrates the coverage of the existing EST collection, with over 50% of protein sequence derived from survey reads matching *Glycine *proteins that are already known. In total, 10,464 of the survey reads were identified as derived from likely conserved protein coding regions (using e <1E-6 BLAST (blastx) hits to the nr database); 41% of the identified protein fragments have no detectable similarity to known soybean protein sequences, giving over 4,000 potential novel soybean protein fragments with similar, conserved protein sequences known from other organisms.

## Discussion

Comparison of 454 survey sequences to previously sequenced BAC clones can reveal regions of multiple-copy sequence and allow approximate quantitation of copy number. Since no bacterial cloning is necessary, a significant advantage of this approach is that repetitive sequences which are refractory to cloning in E. coli [12] can be characterized without a cloning step.

It is possible to use the survey sequences to reconstruct a representative dataset of soybean highly-repetitive sequences *in silico *on a whole-genome scale, because sequences which assemble with 7% genome coverage will almost all be present in multiple copies. Using this method 20,670 multi-copy sequences were found, of which 4,213 are estimated to be present in 100 or more copies per genome. These sequences include transposons, satellites, putative centromeric and telomeric repeats (often in higher-order repeat units) and multi-copy genes such as those for ribosomal RNA. We have collated, curated and annotated these repeat sequences and developed an on-line database where these sequences can be accessed and searched, and we believe they have utility and biological interest in addition to the detection of repeats for genome assembly. For example, since MULEs can be domesticated to perform conserved developmental tasks [31] it is possible the MULEs detected using this survey in soybean will be of broader biological interest.

Exclusion of these multi-copy sequences and low-complexity simple repetitive DNA gives a dataset of "low or single-copy" DNA sequences that can be potentially used to derive genetic markers in subsequent experiments. Agreement with previous Cot measurements [21,22] provides evidence of a lack of bias in genomic sampling using the 454 sequencing procedure, thus it is possible that high-coverage surveys will be able to detect single-copy regions with greater accuracy than current methods.

Of the 20,670 repeats discovered in our survey, an interesting class are the higher-order repeats composed of slightly divergent repeat units of between 30 and 220 nucleotides. This class represents many of the most abundant repeats in soybean [Additional File 1]. Eukaryotic centromeres are typically composed of satellite sequences with a repeat frequency of between 150 and 210 nucleotides, or approximately the amount of DNA required to fold around a nucleosome [32]. Two 92 bp repeats (based on our analysis, the most abundant sequence family in the soybean genome) form a repeat unit of 184 bp, making these sequences a candidate for a centromeric or pericentromeric satellite. Such satellite sequences, while conserved in size, are highly variable in sequence even within a plant species [33] and show more rapid evolutionary change than euchromatic sequences [32,34] – consequently it is expected that soybean repeats show little sequence similarity to those known in Arabidopsis and its relatives [Additional File 1]. In humans [35] and in Arabidopsis [36] centromeric repeats have been shown to consist of higher order arrays, composed of closely related yet divergent nucleosomal repeat monomers. Our short-read sequencing data allows global analysis of such higher-order structures within abundant satellite DNA, and several sequences in Additional File 1 and the repeat database [26] represent such higher order repeat families, producing contigs between 2,500 and 14,000 base pairs in length. Speculatively, therefore, some of these sequences may represent novel centromeric repeats. These relatively large, high-copy-number satellite repeats are difficult to access by other means, and are often not included even in "completed" genomes such as Arabidopsis [33] because of difficulties in obtaining or assembling BAC clones. A detailed catalog of these higher-order repeats is an important product of the survey approach we describe. Knowledge of these higher order sequences provides both a screen for clones containing such problem sequences, and potentially a method to generate more detailed knowledge of tandem repetitive regions such as centromeres or telomeres.

In a genome such as soybean, where substantial EST sequencing has been performed, but the genome itself is not completely sequenced, the genomic survey data can also provide estimates of the copy number of any genes characterized at the molecular level. Copy number of genes is known to affect agronomically relevant traits in soybean, such as allergenicity [37]. In addition to gene copy number estimation, detailed knowledge of repeat sequences, and the ability to screen these sequences from any shotgun genome sequencing dataset, are of significant value to any sequencing and assembly project. While our survey was aimed primarily at investigating repetitive sequences, we also generated some data on partial protein-coding sequences. Most of the sequences we discover with hits to known proteins, but not to known soybean proteins, are likely to represent the regions of incomplete coverage within transcripts partially covered by known ESTs. It is also possible that some of our short sequences are not of sufficient length to generate significant hits. However, some hits from non-plant eukaryotes, bacteria and viruses are seen. These sequences may indicate the presence of a small number of coding sequences in soybean without homology in the completely sequenced plant genomes. We cannot exclude the possibility that our sequences are too short to generate significant scoring alignments with some orthologous plant proteins. It is also possible that these sequences result from microbial DNA contamination, or that homologous proteins exist within, for example Arabidopsis or rice but that these proteins have not been annotated and placed in the nr database. The utility of such a coding region fragment discovery project includes the potential to design microarray probes to coding sequences that may not be present even in detailed EST sequence sets.

## Conclusion

We have developed and validated a method for genomic survey sequencing; a high-coverage, short-read genome survey using 454 pyrosequencing. This method provides no *de novo *assembled sequence, and is not a replacement for conventional shotgun genomic sequencing, or for EST sequencing. However, rapid sequencing of many short genomic fragments gives a clear picture of overall genome composition. Given the much lower cost of the method when compared to Sanger-based whole-genome sequencing or EST sequencing, it can provide a substantial amount of information as a preliminary step to characterize large, unsequenced genomes.

Even much higher coverage sequencing of soybean, using random short reads of the size described here, would be unlikely to allow the assembly of a complete genome sequence. Short sequence fragments, together with the extensive repeats we describe, would cause insoluble difficulties in whole-genome assembly. However, a 454 pyrosequencing genome survey allows the derivation of many types of valuable information, including repeat composition, genome size and genomic copy number. Higher coverage would further increase the value of this type of survey, in particular the coverage of single-copy protein-coding sequences. Ultimately, advances in read length (up to 500 bp or more), and the availability of paired reads, could make possible true whole-genome shotgun sequencing of soybean and other crop plants at greatly reduced cost.

## Methods

### Soybean nuclear genomic DNA isolation

8 g of young trifoliate leaves were taken from soybean cv. Williams, grown under controlled greenhouse conditions in sterile soil. Leaves were ground to coarse powder in N_2_(l), transferred to 20 ml NIB (Modified from Zhang et al [38]; 10 mM Tris, 10 mM EDTA, 100 mM KCl, 500 mM sucrose, 4 mM spermidine and 0.1% β-mercaptoethanol), and placed on ice for 10' with swirling every 1'. The suspension was filtered through 2 layers of Miracloth and 2 layers of cheesecloth, and 1 ml 10% Triton X-100 in NIB added. The suspension was incubated on ice for a further 10' with swirling every 1', then centrifuged at 2,000 g for 15' at 4°C. The supernatant was discarded and the pellet resuspended in 20 ml NIB. After centrifugation for 2' at 100 g, the supernatant was transferred to a fresh tube and the pellet discarded. After centrifugation for 15' at 2,000 g, the supernatant was discarded and the pellet inspected for any green coloration. The centrifugation and resuspension steps were repeated until the pellet was pure white in color. Once free of visible chloroplast contamination, the pellet was resuspended in 10 ml TE (10 mM Tris pH 7.5, 1 mM EDTA). 1 ml of 10% sodium lauryl sulphate was added and 50 mg protease K powder. The resulting suspension was incubated for 48 h at 37°C with slow orbital shaking. 1 ml 3 M sodium acetate pH 5.3 and 10 ml phenol/chloroform/IAA were added, the solution was gently emulsified and centrifuged for 5' at 10,000 g. The aqueous phase was removed and the extraction repeated. 25 ml ethanol was added, the contents mixed and incubated at 20°C for 14 h, followed by centrifugation at 13,000 g for 15'. The pellet was washed twice in 70% aqueous ethanol, resuspended in 100 μl TE, reprecipitated by the addition of 100 μl isopropanol, centrifuged at 13,000 g for 15' and resuspended in 100 μl TE.

### DNA sequencing

DNA sequencing was performed on sheared soybean genomic DNA isolated as above by 454 Life Sciences, Branford, CT at the 454 Sequencing Center using the GS-20 instrument [9]. Two 1.6 million-picowell plates were sequenced, and reads were filtered and trimmed to 5% or fewer marginal calls as described [9]. Further trimming was then performed based on the phred-equivalent quality score for each base (-10 log P(e), [14]). The reads were further trimmed of leading and trailing bases where Q < 10, in order to ensure comparable data to BAC-based surveys [10]. The mean Q value was 26 across the sequences after filtering and trimming, and the longest read was 410 bases and the shortest 35 bases, with a standard deviation of the mean length of 18 base pairs. In the filtered, trimmed sequences, 95% of bases were Q10 or higher, 83% were Q20 or higher. While these quality scores are relatively low by comparison to automated Sanger sequencing of small clones, they are comparable to the levels of quality obtained in whole-genome sample sequencing of the soybean genome using BAC end sequences [10]. Possible contaminants resembling organellar sequences were counted, but not removed, since reads with sequence identity to organelle sequences may be derived from organellar DNA or be genuine genomic sequence with high similarity to the organellar genome. A total of 6,819 reads (0.9%) showed significant (1E-6 BLAST (blastn) hit) identity to a collection of available chloroplast sequences, 958 reads (0.1%) showed a similar level of identity to a collection of mitochondrial sequences. For organellar contaminant estimation, fully assembled soybean chloroplast and mitochondrial sequences were not available; chloroplast and mitochondrial genome sequence from plants including all available soybean data were assembled into a BLAST database in-house. Of the remaining reads, the overall GC content of the sequence was 33%. The full sequence dataset of the soybean 454 genome survey has been deposited at the NCBI Trace Archive, TI range 1732557604-1733276192. Assemblies are available from the authors on request or at their web site [26].

### Detection and quantitation of repetitive sequences

Phrap [19], compiled with the manyreads option on a dual Xeon 2.4 Ghz server with 4 GB DDR2 RAM, with the -ace output option, was used for high throughput assembly of the short read sequences. Parameters were tested to optimize assembly of higher-order repeats. In most cases the default parameters for scores, pentalties, trimming (-trim_qual = 13 -trim_score = 20) were found to be optimal. The assembly of the short reads was found to be very sensitive to the -minmatch parameter. Minmatch values above 14 led to higher-order repeats validated by PCR not being assembled, while values of 12 or less caused the program to crash. Ultimately, 14, the default value, was the value used for the assembly described here. The resultant contigs were either 1) sequences which overlapped by chance, or 2) sequences present in multiple copies per genome. We modeled the probability of generating contigs from sequences which overlap by chance using an implementation of the Poisson distribution developed by Lander and Waterman [20]. The number of contigs expected containing a number of reads *j *is given by equation 1.

(1) $$\begin{array}{l} Ne^{-2c\sigma }{\left(1-e^{c\sigma }\right)}^{j-1}\\ \begin{array}{cc} c=\frac{LN}{G} & \sigma =1-\frac{T}{L} \end{array} \end{array}$$

Where *N *is the number of reads, *L *is the read length, *G *the haploid genome size in base pairs, and *T *the base pair overlap required for contig formation (in this case equivalent to the phrap minmatch parameter, 14). The 'expected' number of reads (from a perfectly non-repetitive genome) was subtracted from the observed number of reads in order to determine the repeated sequences. No contigs containing more than five reads were expected to occur by chance given the depth of coverage of our survey [Table 1].

### Copy number estimation

DNA fragments were matched to the fragment for which copy number is to be determined using BLAT [17]. The number of base pairs matching in BLAT hits with 100% sequence identity was used to provide a minimum copy number, since duplicated genes may have highly similar sequences. Estimated copy number, C, within any sequence window was calculated by equation 2:

(2) $$\begin{array}{c} C=\frac{o}{e}\\ \begin{array}{cc} e=\frac{cw}{L} & c=\frac{LN}{G} \end{array} \end{array}$$

Where *o *represents the observed number of reads matching the sequence window, *e *represents the expected number of reads matching a single-copy sequence window of size *w*, *c *represents coverage, *w *represents window size in base pairs, *N *the total number of reads in the survey, *L *the average read length of the survey in base pairs, and *G *the haploid genome size in base pairs. In this survey, *c *= 0.07 and *L *= 109.5. For the purposes of this study, any region of a clone with an estimated copy number less than one was assigned an estimated copy number of one.

### Assembly of sequences to exemplar BAC sequences using BLAT and BLASTZ

For estimation of quality using assembled reads to the 103 kb exemplar sequence [16] BLAT [17] was used with a 95% identity cutoff (otherwise with default nucleotide options) to identify strongly matching reads. All matching reads were then excluded where the matching block did not extend across 98% or more of the entire read, thereby removing reads that did not match at this identity level across their entire length. Estimated probability of any base being correct was then calculated by dividing the number of matching bases by the number of mismatched bases, plus any bases at the end of the read not included in the matching block, plus the number of matching bases. Percentage of correctly matched bases was given by the correct-base probability multiplied by 100.

For copy number estimation, survey reads were identified as being contiguous with the BAC sequence using BLAT with default parameters, a tile-size of 11 and a minimum score of 30 (this results in a "significant" match criterion of a minimum exact match of two eleven-base tiles with an intervening gap of two or fewer bases, and a minimum percentage match of 90% across the entire block – generally in our experience this is roughly equivalent to a blastn search with e-value cutoff of 1E -20). In the copy number estimation [Fig 2], an alignment was performed using blastz [27] with default options for gap penalties, MSP and gap thresholds, chaining and word size. Reads not producing an alignment matching these criteria were excluded. The Laj applet [29] was then used for visualization for Fig. 2 and for the web site alignment tool.

### BLAST searches

Where not otherwise stated, BLAST [39] programs were used with an e value cutoff of 1E-6, and repetitive sequence filtering on except when matching to repeat databases, where the filter was off. The number of significant hits and alignments (-v and -b options) was limited to 20. Otherwise the parameters were used at default settings.

### Amplification and sequencing of repetitive DNA sequences

DNA was amplified using the PCR in an MJ research DNA Engine thermal cycler (Bio-Rad, Hercules, CA), and reaction conditions were modified to favor amplification of repeats. Reactions were performed in a total volume of 50 ul containing 30 ng/ul Soybean genomic DNA, 1.2 mM MgCl, 0.1 ug/ul BSA, 0.15 mM dNTPs, 0.025 units/ul of Extaq (Takara Mirus Bio, Madison, WI), 0.6 × Ex taq buffer, and 0.05 uM of each oligonucleotide primer. Initial denaturing was at 94 C for 2 min. This was followed by 30 cycles of a 30s denaturing step at 94C, a 40s annealing step at 58C and a 3m extension at 72C. This was followed by a 30 m final extension at 72C.

The primer pairs used were:

MH103 (CATCCATGTTGGTAAGCACCAG) and

MH104 (GGGCATAATAAGGCTTTACACGT),

MH123 (GGTGCAGTTATGGTTTGGGA) and

MH124 (TCTAGAGGTATCATCACTCAAG),

MH155 (TAAAGATGTATTGTCGGAAGATGGGGGC) and

MH156 (TCGAGTTTGGTGCTGTGTTAAATGATTGC).

These primers were designed to amplify segments of Contigs 80285, 80374 and 80369 respectively. The primers were designed completely from sequence derived from assembly of non-cognate small 454 sequence reads. The base quality levels from the 454 sequence assembly had Q values of 40 or greater for all bases underlying the primer.

Plasmid cloning of PCR products was performed using T/A overhang cloning into pGEM-T easy (Promega, Madison, WI). The clones were end-sequenced using BigDye terminator premix (ABI, Foster City, CA) and the vector primers SP6 and T7. Products that failed to clone were end sequenced with the primers used to amplify the product.

### Protein coding sequence detection and annotation

Sequence reads were translated in all six reading frames and resulting putative peptides were matched to the GenBank nr database. Reading frame translations with BLAST (blastp) hits of 1E-6 or lower were considered to be coding sequence fragments. Percentage identity across the matched region, as given by the BLAST output, was then further used to divide the matches into the groups shown in Figure 3.

## Authors' contributions

KS performed analysis of bioinformatics data and comparison of databases and laboratory experiments to validate predicted repeats, created the data displays and helped draft the manuscript. KV developed and implemented assembly and database bioinformatics methods, implemented and performed parallel analysis and annotation of sequences and repeats, and developed the web interface for the database and alignment viewer. MEH conceived the study, design, co-ordination and manuscript, developed and implemented the DNA extraction procedure, assembly and repeat detection, analysis of copy number and the remaining bioinformatics and scripting.

## Supplementary Material

Additional file 1 High abundance repeats. The 40 most abundant higher-order repeat sequences in soybean, as predicted from non-cognate assembly of the short read sequence survey. Click here for file

Additional file 2 Amplification of selected higher-order repeat and retroelement contigs. A negative image of an ethidium bromide-stained agarose gel showing amplification of bands of the expected size. Expected size is given below the band (in base pairs), and size markers are shown at the left hand size (1 kilobase pair intervals). Lane 1: Amplification of soybean genomic DNA with primers designed to Contig 80285, a gag-pol type retroelement, with an expected amplicon size of 4507 bp based on the assembled sequence. Lane 2: Amplification of soybean genomic DNA with primers designed to Contig 80374, a higher-order repeat unit of the STR120 satellite sequence, with and expected amplicon size of 8491 bp. Lane 3: Amplification with primers designed to Contig 80369, another predicted higher-order repeat of the STR120 satellite sequence, with an expected amplicon size of 6739 bp. Click here for file
