# Supplementary material for: Global repeat discovery and estimation of genomic copy number in a large, complex genome using a high-throughput 454 sequence survey
Source: BMC Genomics. 2007 May 24;8:132. doi: 10.1186/1471-2164-8-132 (PMC1894642; doi:10.1186/1471-2164-8-132)
Supplement: Additional file 1 — High abundance repeats. The 40 most abundant higher-order repeat sequences in soybean, as predicted from non-cognate assembly of the short read sequence survey. [file 1471-2164-8-132-S1.pdf]

Supplementary File 1: The 40 most abundant higher-order repeat sequences in soybean, as predicted from assembly of the short read sequence survey.

Data base ID	Length (bp)	Est. % of genome	Best current GenBank hit	Repeat family
80377	13386	0.36	emb|Z26334.1|GMP3X1SAT G.max satellite DNA	SB92 repeat
80376	13092	0.33	emb|Z26334.1|GMP3X1SAT G.max satellite DNA	SB92 repeat
80375	9911	0.26	emb|Z26334.1|GMP3X1SAT G.max satellite DNA	SB92 repeat
80374	8916	0.25	gb|U26701.1|GMU26701 Glycine max satellite STR120-B.1	STR120 satellite
80373	6678	0.23	gb|AF186186.1|AF186186 Glycine max retrovirus-like element Calypso5-1, partial sequence	STR120 satellite and a retroelement
80372	6743	0.23	emb|Z26334.1|GMP3X1SAT G.max satellite DNA	SB92 repeat
80371	9930	0.21	emb|Z26334.1|GMP3X1SAT G.max satellite DNA	SB92 repeat
80370	8197	0.19	emb|Z26334.1|GMP3X1SAT G.max satellite DNA	SB92 repeat
80369	8269	0.16	gb|U26698.1|GMU26698 Glycine max satellite STR120-A.2	STR120 satellite
80368	9309	0.16	gb|AF297983.1|AF297983 Glycine max clone TRS1 tandem repetitive repeat region	SB92 repeat
80367	6325	0.15	previously undescribed retroelement	SIRE
80366	5613	0.14	gb|AF297985.1| Glycine max clone TRS3 tandem repetitive repeat region	SB92 repeat
80365	7401	0.13	gb|AF297985.1| Glycine max clone TRS3 tandem repetitive repeat region	SB92 repeat
80364	3789	0.12	gb|AF297983.1|AF297983 Glycine max clone TRS1 tandem repetitive repeat region	SB92 repeat
80363	5168	0.12	gb|U26699.1|GMU26699 Glycine max satellite STR120-A.3	SB92 repeat
80362	4505	0.12	previously undescribed retroelement	calypso / diaspora
80361	6307	0.12	gb|AF297983.1|AF297983 Glycine max clone TRS1 tandem repetitive repeat region	SB92 repeat
80360	5757	0.12	gb|AF297985.1| Glycine max clone TRS3 tandem repetitive repeat region	SB92 repeat
80359	6040	0.11	unknown rpt sequence	found in soy ESTs

80358	5454	0.11	previously undescribed retroelement	calypso / diaspora
80357	5620	0.11	gb|AF297985.1| Glycine max clone TRS3 tandem repetitive repeat region	SB92 repeat
80356	4775	0.11	emb|Z26334.1|GMP3X1SAT G.max satellite DNA	SB92 repeat
80355	2945	0.11	previously undescribed retroelement	calypso / diaspora
80354	4673	0.11	previously undescribed retroelement	SIRE
80353	2688	0.10	18S ribosomal RNA	rRNA
80352	4832	0.10	previously undescribed retroelement	SIRE
80351	5601	0.10	previously undescribed retroelement	calypso / diaspora
80350	3773	0.09	previously undescribed retroelement	SIRE
80349	5318	0.09	gb|AF297983.1|AF297983 Glycine max clone TRS1 tandem repetitive repeat region	SB92 repeat
80348	3781	0.09	emb|Z26334.1|GMP3X1SAT G.max satellite DNA	SB92 repeat
80347	4451	0.09	previously undescribed retroelement	Calypso
80346	4068	0.09	previously undescribed retroelement	Diaspora
80345	3227	0.09	Previously unknown repeat sequence	
80344	6201	0.09	previously undescribed retroelement	calypso / diaspora
80343	4527	0.09	previously undescribed retroelement	SIRE
80342	4795	0.09	previously undescribed retroelement	calypso / diaspora
80341	3733	0.08	previously undescribed retroelement	calypso / diaspora
80340	3261	0.08	previously undescribed retroelement	calypso / diaspora
80339	5164	0.08	previously undescribed retroelement	calypso / diaspora
80338	4818	0.08	gb|AF297983.1|AF297983 Glycine max clone TRS1 tandem repetitive repeat region	SB92 repeat
80337	3649	0.08	previously undescribed retroelement	calypso / diaspora
