# Supplementary material for: Global repeat discovery and estimation of genomic copy number in a large, complex genome using a high-throughput 454 sequence survey
Source: BMC Genomics. 2007 May 24;8:132. doi: 10.1186/1471-2164-8-132 (PMC1894642; doi:10.1186/1471-2164-8-132)
Supplement: Additional file 2 — Amplification of selected higher-order repeat and retroelement contigs. A negative image of an ethidium bromide-stained agarose gel showing amplification of bands of the expected size. Expected size is given below the band (in base pairs), and size markers are shown at the left hand size (1 kilobase pair intervals). Lane 1: Amplification of soybean genomic DNA with primers designed to Contig 80285, a gag-pol type retroelement, with an expected amplicon size of 4507 bp based on the assembled sequence. Lane 2: Amplification of soybean genomic DNA with primers designed to Contig 80374, a higher-order repeat unit of the STR120 satellite sequence, with and expected amplicon size of 8491 bp. Lane 3: Amplification with primers designed to Contig 80369, another predicted higher-order repeat of the STR120 satellite sequence, with an expected amplicon size of 6739 bp. [file 1471-2164-8-132-S2.pdf]

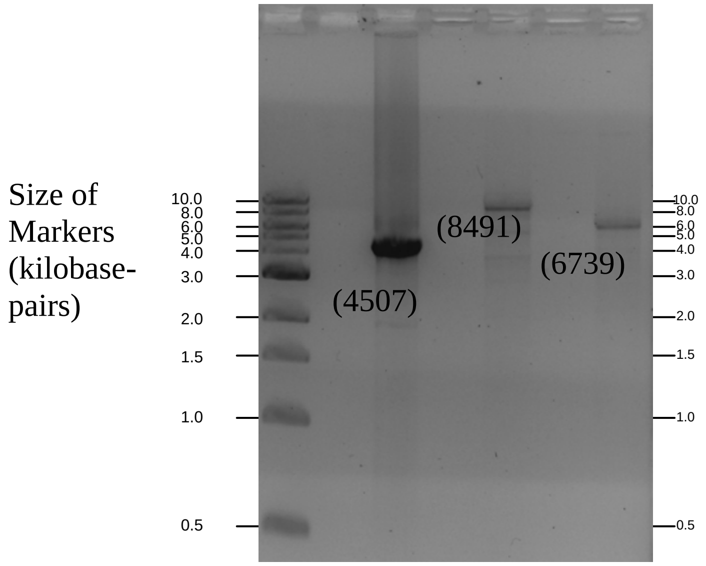

Supplementary File 2
Amplification of selected higher-order repeat and retroelement contigs.

A negative image of an ethidium bromide-stained agarose gel showing amplification of bands of the expected size. Expected size is given below the band (in base pairs), and size markers are shown at the left hand size (1 kilobase pair intervals). Lane 1: Amplification of soybean genomic DNA with primers designed to Contig 80285, a gag-pol type retroelement, with an expected amplicon size of 4507 bp based on the assembled sequence. Lane 2: Amplification of soybean genomic DNA with primers designed to Contig 80374, a higher-order repeat unit of the STR120 satellite sequence, with and expected amplicon size of 8491 bp. Lane 3: Amplification with primers designed to Contig 80369, another predicted higher-order repeat of the STR120 satellite sequence, with an expected amplicon size of 6739bp.
